# Implementation of Online Mindfulness With Peer Mentoring for Parent and Sibling Carers of People With Intellectual and Developmental Disabilities

**DOI:** 10.1111/jir.70057

**Published:** 2025-10-20

**Authors:** Caitlin A. Murray, Nikita K. Hayden, Alex Gordon‐Brown, Samantha Flynn, Clare Bonetree, Andrew Harper, Clare Kassa, David Mahon, Catherine McGee, Richard P. Hastings

**Affiliations:** ^1^ Centre for Research in Intellectual and Developmental Disabilities (CIDD) University of Warwick Coventry UK; ^2^ School of Education, University of Sheffield; iHuman University of Sheffield Sheffield UK; ^3^ Family Fund York UK; ^4^ Sibs Keighley UK; ^5^ Foundation for People With Learning Disabilities London UK; ^6^ Contact a Family London UK; ^7^ Intellectual Disabilities Research Institute (IDRIS), University of Birmingham School of Social Policy and Society, Edgbaston Birmingham UK

**Keywords:** family carers, implementation, intervention, mindfulness, parents, peer support, siblings

## Abstract

**Background:**

There is promising evidence for the adaptation of online mindfulness interventions for parent carers of individuals with intellectual and developmental disabilities by including supplementary peer support sessions. However, there remain questions about wider implementation beyond the research setting and the inclusion of more diverse populations of family caregivers, including adult siblings and family carers who less typically receive support and are often under‐represented in research.

**Method:**

One hundred and one family carers (*n* = 58 parents, *n* = 43 adult siblings) were provided with access to Be Mindful (an online mindfulness intervention) with additional telephone peer mentor support. Participants were asked to complete baseline and follow‐up questionnaires before and after the intervention in a pre‐post pre‐experimental design, and engagement with the intervention and peer support was examined.

**Results:**

Recruitment was successful in targeting more diverse groups, including adult siblings. Intervention completion was low overall (*n* = 37). Parent and sibling carers made differing levels of progress with the intervention and peer support calls, although 81.8% of those who completed the intervention before the end of the project had also received all three support calls. Preliminary follow‐up data, though with low retention, indicated improvements in psychological wellbeing for family carers over time.

**Conclusions:**

The intervention and additional telephone‐guided support were received well by family carers of people with intellectual and developmental disabilities, although further work is needed to determine the feasibility of future implementation.

A recent systematic review and meta‐analysis concluded that mindfulness‐based interventions may be effective in decreasing stress, anxiety and depression in parents of children with intellectual and developmental disabilities (Chua and Shorey 2022). Additionally, telehealth delivery methods can be effective for the delivery of support for parents of children with neurodevelopmental conditions (Kelson and Dorstyn 2023). Bringing both content and delivery approach together, there is some evidence that self‐directed mindfulness online support can help to improve parental wellbeing. Flynn et al. (2020) conducted a feasibility randomised controlled trial (RCT) with 60 parental caregivers of children and adults with intellectual disabilities who accessed the Be Mindful, a low‐cost, referral‐only online mindfulness programme which has been developed to include all the elements of Mindfulness‐Based Cognitive Therapy (MBCT) by MBCT experts. It has been shown to be an acceptable, accessible intervention with reductions in stress, anxiety and depression post‐intervention for self‐referred participants and in the context of an RCT (Krusche et al. 2013; Querstret et al. 2018). In the Flynn et al. (2020) study, parental caregivers were allocated to either receive Be Mindful as usual with no additional support or Be Mindful with additional peer mentor support sessions from other parental caregivers. Preliminary findings indicated that wellbeing, as measured using the Warwick Edinburgh Mental Well‐being Scale (WEMWBS; Tennant et al. 2006), increased for parents in both trial arms, with limited indication of a more positive impact for the peer support group.

The Flynn et al. (2020) study showed promising evidence for the positive impact of an online mindfulness programme for parental caregivers of children and adults with intellectual disabilities. Using an existing online, asynchronous programme has potential advantages in terms of access (e.g., greater flexibility and reduced demands for travel and childcare), but there are key questions that need to be considered in relation to the wider implementation of support for family caregivers. First, family carers are not always parents; adult siblings often have caring responsibilities for their siblings and may also benefit from wellbeing support (Hayden et al. 2023). Second, in practice, ensuring that family caregivers can access and then engage with a support programme may be a challenge. Third, there are often barriers to reaching families experiencing socio‐economic deprivation, ethnic minority families and male family members, leading to an under‐representation of these groups in research (Bencheva et al. 2024; Blacher et al. 2020). The main aim of the current study was to implement the peer‐supported Be Mindful programme with a community‐based implementation partner for a more diverse population of family caregivers of children and adults with intellectual and developmental disabilities, including adult siblings and family carers who are less typically likely to receive support and are often under‐represented in research.

## 1 Method

### 1.1 Participants

Participants were adult sibling and parent carers (referred to here collectively as family carers) who responded to targeted recruitment advertisements via charity partners, support groups and social media and completed a short form to determine eligibility for the project. To reach a more diverse population, recruitment was staged to first focus on adult siblings, father and brother support groups, and families in contact with organisations working with those experiencing socio‐economic deprivation, and in areas with higher ethnic minority populations compared to the general UK population, before moving to a wider recruitment of family carers. Family carers were excluded only if they had previously used Be Mindful. One hundred and sixty‐five family carers completed the screening form, with 101 family carers completing the baseline questionnaire and taking part. Table 1 provides a full summary of family carer demographics.

**TABLE 1:** Summary of socio‐demographic characteristics.

Demographics	Parents (*n* = 58)		Siblings (*n* = 43)	
	*N*	%	*N*	%
**Family carer**				
Gender				
Male	9	15.3	2	4.7
Female	49	84.7	41	95.3
Mean age (SD)	43.5 (SD 8.1)	—	43.2 (SD 13.2)	—
Ethnicity				
White British	47	81.0	35	81.4
Asian/Asian British	5	8.6	4	9.3
White other	1	1.7	1	2.3
Black British	2	3.4	1	2.3
Any other ethnic background	3	5.2	2	4.7
Educational level				
No qualifications	3	5.2	0	0
Secondary/high school education^a^	16	27.6	3	7.0
Other higher education below degree level	17	29.3	7	16.3
Degree (bachelors) or higher	20	34.5	33	76.7
Employment				
Currently working	23	39.7	27	41.9
On long‐term leave (e.g., maternity, paternity and sick)	0	0	1	2.3
Full‐time carer	24	41.4	3	7.0
In education	1	1.7	4	9.3
Not working but looking for work	1	1.7	1	2.3
Not working and not looking for work	8	13.8	6	14.0
Country				
England	47	81.0	37	86.0
Scotland	6	10.3	5	11.6
Wales	4	6.9	1	2.3
Northern Ireland	1	1.7	0	0
Below UK median weekly household income	38	65.5	24	55.8
Living in most deprived 10% of neighbourhoods based on indices of multiple deprivation (IMD)	6	10.3	3	7.0
**Person with intellectual and/or developmental disability**				
Gender				
Male	36	62.1	26	60.5
Female	19	32.8	16	37.2
Mean age (SD)	11.4 (SD 8.3)	Range 2.5–54 years	39.72 (SD 14.49)	Range 10.0–61.0 years
Living with family member				
Yes	56	96.6	7	16.3
No	2	3.4	36	83.7
Diagnosis as reported by family carer				
Autism	40	70.0	29	67.4
Learning disability	32	55.2	36	83.7
Down syndrome	2	3.4	4	9.3
Other genetic syndrome	17	29.3	10	23.3

^a^ ‘Secondary/high school education’ here is within the UK context so refers to completion of any General Certificate of Secondary Education (GCSEs) or equivalent (exams at age 16) and/or leaving qualifications such as Advanced Level qualifications (A‐levels) or equivalent (exams at age 18).

### 1.2 Design

The study is a pre‐experimental pretest–posttest design focused on implementation.

### 1.3 Be Mindful With Additional Peer Support

Be Mindful is an online mindfulness programme, with 10 online sessions based on mindfulness‐based cognitive therapy (MBCT), 12 assignments to practice in daily life, six course handouts and supporting emails throughout (see Table S1). The programme can be accessed on any device with a web browser and an internet connection. The provision of additional peer mentor support to provide three peer support calls encouraging and supporting participants in their Be Mindful progress was adapted from Flynn et al. (2020), where there was a positive impact on wellbeing with the inclusion of the additional peer support compared to Be Mindful alone. Parent and sibling peer mentors were recruited to the paid role through partner organisations and were matched with family carers by the community‐based implementation partner based on role (i.e., parent and sibling) and availability, with the age of the person they cared for (i.e., child and adult) considered where possible. Peer mentors followed an updated coproduced manual (Flynn et al. 2020), attended a 1.5‐day virtual training workshop, and completed Be Mindful themselves. Peer mentors were supervised by the community‐based implementation partner and had access to WhatsApp groups to facilitate their own peer support. The peer support manual was updated to reflect the inclusion of sibling carers. All family carers were offered three 30‐min telephone calls from a peer mentor in addition to free access to Be Mindful, with peer mentors contacting their peer mentees to arrange the three sessions (see Flynn et al. 2020).

Ten parent carers (all mothers) and five adult siblings (four sisters and one brother) were recruited into the paid peer mentor role through targeted recruitment via charity partners. All were fully trained and completed the online Be Mindful course themselves. Three parent mentors completed the training (two of the three completed Be Mindful) but withdrew before taking on any mentees as they were unable to commit to the role at the time. The four sibling mentors who remained each supported between 7 and 13 sibling carers; the sibling mentor who was unable to continue in the role supported three sibling carers. The five parent mentors who remained supported between 8 and 11 parent carers. One parent mentor who withdrew supported five parent carers, and the final parent mentor who was recruited later in the project provided support to three family carers.

### 1.4 Measures

Participants provided demographic data about themselves, their family and the person for whom they cared (Table 1). The primary outcome measure for the study was psychological wellbeing as measured by the short Warwick‐Edinburgh Mental Well‐being Scale (seven items; SWEMWBS; Stewart‐Brown et al. 2009). The internal consistency of this measure at baseline was α = 0.77. Participants also reported on their perceptions of their psychological distress (six items; baseline α = 0.86; Kessler Psychological Distress Scale [K6], Kessler et al. 2002), family functioning (five items; Family APGAR scale; Smilkstein 1978; α = 0.84 at baseline), and a single‐item question on life satisfaction rated from 0 (*not at all satisfied*) to 10 (*completely satisfied*). Data were available on the progress and completion of Be Mindful and the number of peer mentoring calls completed.

### 1.5 Procedure

Family carers completed the baseline questionnaire and had their first peer mentor call before starting Be Mindful. Two additional peer mentoring calls were scheduled while completing Be Mindful, and a short follow‐up questionnaire was sent to family carers 12 weeks following the start of the intervention. Participants completed the questionnaire online or by telephone and received email reminders, telephone calls and a project newsletter to encourage completion of the follow‐up questionnaire. Shortened versions of the follow‐up questionnaire were also made available following several reminders. Family carers did not receive a monetary incentive to complete either the baseline or follow‐up questionnaires. Ethical approval was granted by the University of Warwick's Humanities and Social Sciences Research Ethics Committee (reference 101/21‐22).

### 1.6 Data Analysis

Baseline characteristics of the family carers and engagement in the intervention are reported in Tables 1, 2, 3. Pre‐intervention and post‐intervention scores are reported in Table 4 with paired sample *t*‐tests, and effect sizes reported using Cohen's *d* with Dunlap's correction for repeated measures (Dunlap et al. 1996). Sample size prevented the exploration of whether any change in wellbeing varied depending on whether family carers were more or less engaged with the intervention or had previous experience of mindfulness. As an exploratory analysis, one‐way analysis of covariance (ANCOVA) was used to explore statistically whether differences in post‐intervention wellbeing scores between parents and siblings were meaningful after controlling for pre‐intervention scores. We focused only on wellbeing for this analysis given that the sample size was reasonable for this comparison (*n =* 36; 21 siblings and 15 parents), and wellbeing was the primary outcome of interest.

## 2 Results

One hundred and sixty‐five family carers expressed interest in participating in the project. Of these, 89 were mothers, 10 fathers, 4 brothers and 60 sisters. All completed a screening form, and 101 family carers were eligible, consented to participate and completed the baseline questionnaire. The recruited sample included 58 parent carers (49 mothers and 9 fathers) and 43 sibling carers (2 brothers and 41 sisters). Six participants (3 parents and 3 siblings) withdrew from the project between baseline data collection and follow‐up. Of the 95 remaining participants, 36 completed measures at follow‐up: 21 siblings (48.8% return rate) and 15 parents (25.9% return rate). Twelve participants completed only the SWEMWBS at follow‐up (as a minimum dataset).

Engagement with Be Mindful and the peer mentoring calls is summarised in Tables 2 and 3. In total, 37 participants (23 siblings and 14 parents) fully completed Be Mindful before the end of the project. Four completed within the intended time frame (4 weeks). When completed, the mean time taken to complete Be Mindful was 71 days (median = 64, range = 31–266 days). For peer mentoring calls, 49 family carers (19 parents and 30 siblings) received all three calls; 30 of these participants completed the intervention. Nine family carers (5 parents and 4 siblings) received two calls, 12 received one call (9 parents and 3 siblings) and 19 (13 parents and 5 siblings) received none. Two family carers who received two calls completed Be Mindful, as did one family carer who received no calls. Of those who received no peer mentoring calls, 3 family carers withdrew prior to arranging a call with their peer mentor, 11 family carers did not respond to their peer mentor, and 5 family carers arranged their first call but cancelled or did not pick up and did not respond to further contact. For one of these five family carers, this was due to scheduling conflicts with their assigned peer mentor; they were matched with a new peer mentor but still did not respond.

**TABLE 2:** Engagement with intervention.

	Parents (*n* = 58)		Siblings (*n* = 43)		Total (*N* = 101)	
	*N*	%	*N*	%	*N*	%
**Previous experience of mindfulness**						
Yes	21	36.2	24	55.8	45	44.6
No	37	63.8	19	44.2	56	55.4
**Be Mindful progress**						
Did not start Be Mindful	11	19.0	1	2.3	12	11.9
Stopped after introduction	10	17.2	2	4.7	12	11.9
Stopped after Week 1	7	12.1	3	7.0	10	9.9
Stopped after Week 2	9	15.5	6	14.0	15	14.0
Stopped after Week 3	4	6.9	4	9.3	8	7.9
Stopped after Week 4	3	5.2	4	9.3	7	6.9
Fully completed Be Mindful	14	24.1	23	53.5	37	36.6
**Peer mentor calls progress**						
Did not respond to peer mentor invitations	8	13.8	3	7.0	11	10.9
Withdrew prior to calls	1	1.7	1	2.3	2	3.0
Arranged call but no answer/further response	4	6.9	1	2.3	5	5.0
Had one peer mentoring call	9	15.5	3	7.0	12	11.9
Had two peer mentoring calls	5	8.6	4	9.3	9	8.9
Had three peer mentoring calls	19	32.8	30	69.8	49	48.5
Missing data	12	19.0	1	2.3	13	12.9

**TABLE 3:** Number of peer mentor calls and progress in Be Mindful.

		Progress in Be Mindful							Total
		Did not start	Stopped after introduction	Stopped after Week 1	Stopped after Week 2	Stopped after Week 3	Stopped after Week 4	Fully completed Be Mindful	
Number of Peer mentor calls	0	7	7	2	1	0	0	1	18
	1	1	0	3	5	2	1	0	12
	2	1	2	2	1	1	0	2	9
	3	0	1	1	7	5	5	30	49
	Missing	3	2	2	1	0	1	4	13
Total		12	12	10	15	8	7	37	101

Twenty‐four (66.7%) of those who completed the follow‐up survey had fully completed Be Mindful, two family carers had not started or not progressed beyond the introduction, and the remaining 10 were between Week 1 and Week 4 when they stopped Be Mindful. Twenty‐six (72.2%) of those who completed the follow‐up survey had received all three peer mentoring calls. Table 4 presents a full summary of the outcome measure scores at baseline and for those with both pre‐ and post‐intervention data. Paired samples *t*‐tests indicated an increase in psychological wellbeing scores (*t*(34) = −4.78, *p* < 0.001, Cohen's *d* with Dunlap's correction = 0.91), a decrease in psychological distress (*t*(23) = 4.11, *p* < 0.001, Cohen's *d* with Dunlap's correction = 0.72), and an increase in life satisfaction (*t*(23) = −2.99, *p* = 0.006, Cohen's *d* with Dunlap's correction = 0.62) for those participants with pre‐ and post‐intervention data. No significant change over time was found for perceived family functioning. Exploratory analysis using a one‐way ANCOVA did not find any significant differences between parents and siblings for post‐intervention scores of psychological wellbeing, controlling for pre‐intervention wellbeing (*F*_1, 33_ = 0.34, *p* = 0.56, partial η^2^ = 0.01).

**TABLE 4:** Pre‐ and post‐intervention outcomes for core measures using paired sample *t*‐tests.

	Overall baseline data	Pre‐ and post‐complete data, mean (SD)	*t* (95% CI)	*p*	Effect size
Psychological wellbeing^a^					
Baseline	19.29 (2.42)	18.98 (2.24)	—	—	
Follow‐up	—	21.89 (3.77)	−4.78 (−4.15, −1.67)	< 0.001**	0.91
Psychological distress^b^					
Baseline	10.90 (4.84)	10.42 (5.12)	—	—	
Follow‐up	—	6.75 (5.02)	4.11 (1.82, 5.51)	< 0.001**	0.72
Life satisfaction^c^					
Baseline	6.38 (1.95)	6.08 (2.32)	—	—	
Follow‐up	—	7.42 (1.91)	−2.99 (−2.25, −0.41)	0.006**	0.62
Perceived family functioning^d^					
Baseline	4.90 (2.73)	4.61 (2.79)	—	—	
Follow‐up	—	5.30 (2.88)	−1.30 (−1.81, 0.41)	0.207	0.24

^a^ Psychological wellbeing measured by SWEMWBS where higher scores indicate higher positive mental wellbeing (follow up *n* = 36).

^b^ Psychological distress measured by K6 where higher scores indicate higher distress (follow up *n* = 24).

^c^ Higher scores indicate higher overall life satisfaction (follow up *n* = 24).

^d^ Measured by the APGAR scale where higher scores indicate higher perceived family functioning (follow up *n* = 24); all – effect size measured by Cohen's *d* with Dunlap correction for repeated measures design.

** Statistically significant.

## 3 Discussion

We implemented an online programme with peer mentoring for both parent and sibling carers by working together across voluntary organisations in the United Kingdom to reach wider groups of family carers. Over 100 family carers were provided with access to the online Be Mindful programme. The recruitment process was broadly successful, with 76 of the 101 participants reached through targeted recruitment focused on adult siblings, father and brother support groups, and families in contact with organisations working with those experiencing socio‐economic deprivation, and in areas with higher ethnic minority populations compared to the general UK population. For example, 61.4% of family carers in the study reported a weekly household income below the UK median, with 25.7% of family carers indicating they were finding it ‘quite’ or ‘very’ difficult to manage financially. Recruitment and engagement of adult siblings was a particular strength. Despite the targeted recruitment approach, the numbers of fathers and brothers engaged in both the peer mentoring role and with Be Mindful were low. This also meant that for men, matching on gender for mentoring was not possible as there was only one brother and no fathers in the peer mentor role. The Be Mindful online intervention is currently only available in English, and there was no capacity to provide peer mentoring calls in additional languages.

In terms of implementation, 36.6% of participants (*n* = 37; 23 siblings and 14 parents) fully completed Be Mindful before the end of the project. The mean time taken to complete Be Mindful was longer than the intended timeframe of 4 weeks but in line with timeframes reported in Flynn et al. (2020). For peer mentoring calls, 48.5% of participants (*n* = 49; 19 parents and 30 siblings) received all three calls. Thirty of these participants completed the intervention; 81.8% of those who completed the intervention before the end of the project had also received all three calls, indicating that those who were more engaged with calls were more likely to progress further with the intervention. Overall, however, these data suggest that for most family carers the programme was not completed as intended, and further work is needed to explore reasons for disengagement and difficulties with completion to determine feasibility of future implementation.

Parent carers had a reduced engagement rate compared to adult siblings. Adult siblings were more likely to be supporting their adult sibling and less likely to be living with their sibling, while parent carers were more likely to be caring for and living with a younger child. The sibling group had a higher education level compared to the parent group and fewer described themselves as full‐time carers. These factors may have meant that parents were experiencing more day‐to‐day, acute challenges and barriers to completing Be Mindful. Although many adult siblings were also caring for their own families in addition to their sibling, there may be key differences between the parents' and adult siblings' day‐to‐day responsibilities and life stages in terms of their family member with an intellectual and/or developmental disability.

Our findings may also inform future recruitment and support of peer mentors. Parent mentors, in particular, may need to be over‐recruited to account for changes in family circumstances that prevent peer mentors from continuing in their role, either temporarily or permanently. Missing data on call records was increased for parent mentors, possibly indicating the challenges parent carers experienced balancing a paid role and their own carer responsibilities. Further understanding and support for peer mentors would be necessary to ensure this is a mutually beneficial role for family carers and those they are supporting, and to reduce and address barriers for family carers taking on a paid support role. The interest in the peer mentor roles was far greater than expected, indicating a desire for paid work that is accessible, flexible and values and draws on family carer experiences. This is also an area for future development, perhaps with more formal skills development available to mentors throughout the process.

We found an increase in psychological wellbeing and decreased psychological distress for family carers who completed outcome measures at follow‐up. However, response rates were very low and limited to the sample who engaged more with Be Mindful and peer support. In an exploratory analysis, no significant differences in post‐intervention wellbeing (after controlling for pre‐intervention scores) were found between parents and siblings. Tentatively, this may indicate that the programme is appropriate and benefits both siblings and parents. Although encouraging, these data must be treated with caution. The outcome data were analysed for pre‐post change only, as there was no comparison group for this implementation study. To build on Flynn et al. (2020) and the current paper, larger scale, appropriately powered trials are required to be able to detect meaningful differences and establish the intervention as evidence‐based. It was also clear that gathering ongoing evaluation data for implementation projects focused on evidence‐based programmes may be challenging. Informal feedback from family carers suggested that there was some confusion between the Be Mindful automatic reminder emails and those from the researchers; meaning some family carers may have completed follow‐up questions and provided feedback within the Be Mindful programme itself, but not responded to researcher requests.

## Ethics Statement

Ethical approval was granted by the University of Warwick's Humanities and Social Sciences Research Ethics Committee (reference 101/21‐22).

## Conflicts of Interest

D.M. recruited and employed the peer mentors through their role at the Foundation for People with Learning Disabilities. The other authors have nothing to report.

## Supporting information


**Table S1:** Be Mindful online intervention content.

## Data Availability

The data that support the findings of this study are available on request from the corresponding author. The data are not publicly available due to ethical restrictions.
