# Supplementary material for: Implementation of Online Mindfulness With Peer Mentoring for Parent and Sibling Carers of People With Intellectual and Developmental Disabilities
Source: J Intellect Disabil Res. 2025 Oct 20;70(3):337–44. doi: 10.1111/jir.70057 (PMC12872373; doi:10.1111/jir.70057)
Supplement: Supplementary file 1 — Table S1: Be Mindful online intervention content. [file JIR-70-337-s001.docx]

**Supplementary Table 1**

*Be Mindful online intervention content*

| **Week/session** | **Content** | **Homework** |
| --- | --- | --- |
| Getting started | Registration; introduction to course; completion of Stress, Anxiety, and Depression assessment | N/A |
| **Week 1 – Stepping out of automatic pilot** | | |
| Session 1 | Body scan; being mindful doing routine activities; mindful eating | Practice body scan |
| Session 2 | Dealing with barriers | N/A |
| **Week 2 – Reconnecting with body and breath** | | |
| Session 1 | Mindful breathing | Practice mindful breathing; keeping an Event Awareness Journal; practice moving mindfully |
| Session 2 | Physical barometer | N/A |
| **Week 3 – Working with difficulties** | | |
| Session 1 | Breathing space; sitting meditation | Practice breathing space and sitting meditation |
| Session 2 | Thoughts are just thoughts | N/A |
| **Week 4 – Mindfulness in daily life** | | |
| Session 1 | Preparing for stress; reflection on stress strategies | Practice activity awareness, breathing space, and action step; stress strategies |
| Session 2 | Mindful walking | N/A |
| Going forward | Additional resources; completion of Stress, Anxiety, and Depression assessment; completion certificate | N/A |
